# Functional Bioglass—Biopolymer Double Nanostructure for Natural Antimicrobial Drug Extracts Delivery

**DOI:** 10.3390/nano10020385

**Published:** 2020-02-22

**Authors:** Irina Negut, Laura Floroian, Carmen Ristoscu, Cristian N. Mihailescu, Julia Claudia Mirza Rosca, Tatiana Tozar, Mihaela Badea, Valentina Grumezescu, Claudiu Hapenciuc, Ion N. Mihailescu

**Affiliations:** 1 National Institute for Lasers, Plasma and Radiation Physics, Măgurele 077125, Romania; negut.irina@inflpr.ro (I.N.); carmen.ristoscu@inflpr.ro (C.R.); cristi.mihailescu@inflpr.ro (C.N.M.); tatiana.alexandru@inflpr.ro (T.T.); valentina.grumezescu@inflpr.ro (V.G.); hapenciuc.claudiu@inflpr.ro (C.H.); 2 Automation Dept, Faculty of Electrical Engineering and Computer Science, Transilvania University of Brasov, Brasov 500036, Romania; lauraf@unitbv.ro; 3 University of Las Palmas de Gran Canaria, Campus Universitario de Tafira, 35017 Las Palmas de Gran Canaria, Las Palmas, Spain; julia.mirza@ulpgc.es; 4 Fundamental, Prophylactic and Clinical Specialties Department, Faculty of Medicine, Transilvania University of Brasov, Brasov 500036, Romania; badeamihaela1973@gmail.com

**Keywords:** drug release, antimicrobial effect, implant protection, bioactive coatings

## Abstract

Aseptic loosening and periprosthetic infections are the main causes of implant failure. Strategies to mitigate this drawback are therefore mandatory to avoid primary and revision replacement surgeries. A functional bioapatite–biopolymer double nanostructure fabricated by matrix-assisted pulsed laser evaporation to prevent infection of orthopedic and dental implants could promote osseointegration and ensure controlled delivery of natural antimicrobial drugs. The synthesized nanostructure consists of two overlapping layers, the lower from a biocompatible polymer for anticorrosive protection, and the upper of bioactive glass incorporating antimicrobial plant extract, acting as a potential drug delivery system. Morphology, composition, adherence, ability for drug delivery and biological properties (cytotoxicity and antimicrobial effect) were studied. Structures proved compact and stable, conserving a remarkable drug delivery ability for more than 21 days, i.e., enough to ensure long-term microbes’ eradication.

## 1. Introduction

Orthopedic implants are nowadays widely used, but their success can be hindered by infection and poor osseointegration, the two primary causes of implant failure and revision surgeries [1]. 

Given the pathophysiological diversity of the osseointegration process, a particular attention was focused towards the development and evaluation of composite materials for a better osseointegration and bone regeneration. Composite materials based on polymers and bioglasses attracted increased attention due to the potential of producing new materials with improved and combined characteristics of both components via a synergistic effect [2,3,4,5]. Due to their similarities to bone composition and mineral structure but also to the promotion of bone formation, some ceramics and bioglasses display advantageous abilities for scaffolds applications [6]. One discovers, however, limitations to general use of these materials, such as inadequate mechanical properties and a fast degradation rate.

Concomitant with the implant introduction into the body, locally generated microbial infections can occur, as a result of bacteria attachment to the implant surface and subsequent biofilm formation at the site of implantation. A significant stage in implant-associated infection prophylaxis is antibiotic therapy. The choice of antibiotic, form of application and administration time remain issues that are frequently debated in orthopedic surgery [7,8,9,10]. The need for effective antibiotics in bone infections such as aminoglycosides is in contrast with the severe toxicity when applied systemically. In view of these toxic side-effects, appropriate drug doses can be effectively administered by applying them directly to the target site [11,12]. Indeed, the release of drugs to a specific location of the human body is very important since it increases the therapeutic efficiency and ensures optimum drug concentration, under the toxicity threshold. To circumvent infections, attempts were made by: (i) directly imbibe implants with antibiotics, (ii) immobilize an antimicrobial agent in a matrix capable of binding to various surfaces, and/or (iii) construct different composite coatings with antimicrobial active substances [13,14,15,16]. 

Many research teams [12,17,18,19,20] investigated drug delivery systems in form of composite coatings for use in orthopedic or dental implants or prosthesis. Thus, the efficacy of polystyrene-methylmethacrylate films doped with rifampicin, doxycycline and clarithromycin against biofilm formation was studied by assessing methicillin susceptibility and methicillin resistance against *S. aureus* strains for up to 21 days [21]. Nevertheless, coatings containing antibiotics present a number of limitations: a rather low control of drug release, limited area of applications, and alteration of physical-chemical properties of implant due to coverage with passive layers. 

In addition, this approach does not push forward the osseointegration process and does not prevent corrosion of the implant in contact with physiological fluids and further release of metal ions in the body. The use of antibiotic-containing bone cements requires a subsequent surgical removal of cement after the drug has been released, a procedure that implies additional risks: pain, anesthetic complications, and supplementary costs [22]. 

As the common and excessive therapeutic strategy for persistent infections (which represent frequent causes of failure) in endodontics, periodontal surgery and implantology [23,24] still requires antibiotics, common infectious pathogens developed resistance which motivates the search for new therapeutic strategies [25]. To this eventuality, some studies evaluated the potential benefits of different medicinal plants [26]. The use of plant extracts in biomaterial field is gaining popularity because of distinct advantages over classical drugs: superior biocompatibility, availability, good antimicrobial and tissue regenerative activity, and, fortunately, lower side-effects. 

This study focuses on the fabrication by matrix-assisted pulsed laser evaporation (MAPLE) of a two-layered structure on a metallic implant with the double role of natural antimicrobial drug delivery system, and efficient hamper against difficulties related to implant insertion into the human body: slow osseointegration of the bioinert metal implant, corrosion of the implant in contact with physiological fluids, and release of metal ions in the body.

## 2. Materials and Methods 

### 2.1. Materials 

Chemicals, namely chloroform (CHCl_3_), analytical grade acetone (C_6_H_6_O), ethanol (C_2_H_5_OH), and reagents required for preparation of the simulated body fluid (SBF), such as NaCl, NaHCO_3_, KCl, K_2_HPO_4_·3H_2_O, MgCl_2_·6H_2_O, HCl, CaCl_2_, Na_2_SO_4_ and (CH_2_OH)_3_CNH_2_ were purchased from Sigma-Aldrich Chemie GmbH (Germany).

Two (1 × 1) cm^2^ area plates from titanium 4th grade (Ti) and 316L medical grade stainless steel (SS) were utilized as deposition substrates. SS composition was: 64.26 wt% Fe, 18.51 wt% Cr, 12 wt% Ni, 2.13 wt% Mo, 1.44 wt% Mn, 0.58 wt% Cu, 0.56 wt% Si, 0.0265 wt% C, 0.0036 wt% S and smaller concentration of other elements. Prior to deposition, the plates were machined by mechanical polishing to a roughness within 2–4 μm and cleaned with ethanol and deionized water in an Elma X-Tra 30 H ultrasonic bath. 

Bioactive glasses (BG) are degradable, synthetic materials containing key elements and molecules which boost osteogenesis. They have been in use for nearly 50 years [27,28] in the orthopedics field due to their ability to convert and built on surface an apatite layer during dissolution, like hydroxycarbonated apatite, which promotes osteogenesis. BG are based on an amorphous mixture of oxides (SiO_2_-Na_2_O-K_2_O-CaO-MgO-P_2_O_5_). The chosen BG contains 56.5% SiO_2_, 15% CaO, 11% Na_2_O, 8.5% MgO, 6% P_2_O_5_, and 3% K_2_O (in wt%). It is fabricated following the protocol described in Refs. [29,30]. Poly(methyl methacrylate)—PMMA, (C_5_H_8_O_2_)n, is a biopolymer widely used in both medical applications [31] and in some optical systems (contact lens as transmit light up to 93% [32]) or the flat panel industry [33]. It is also used as denture base material due to its high stability in the physiological environment, and ease of handling and repair [34]. 

Neem (*Azadirachta indica*, family *Meliaceae*) is an Indian medicinal plant used in traditional Ayurveda treatment of various infections. It is recommended for dental and oral hygiene (chewing Neem sticks is still the most common method of cleaning the mouth in Indian rural population) [35]. It was shown that Neem exhibits anti-infective, anti-inflammatory, antioxidant, anticariogenic, or immunomodulatory activities, which recommend it for endodontics and periodontal surgery [36]. There exists evidence of the antibacterial effectiveness of Neem extracts on the frequent microorganisms causing dental infections: *Staphylococcus aureus*, *Streptococcus mutans*, *Candida albicans*, or *Enterococcus faecalis*. [37,38,39]. A total of 2% aqueous extract of Neem chewing sticks inhibited in vitro the growth of *Staphylococci, Streptococci* and *E. coli* [40]. Ethanolic extract of Neem leaf demonstrated inhibitory effect on *Methicillin-resistant Staphylococcus aureus* biofilm by decreasing its ability to form large clusters [41]. The methanolic extract of Neem proved robust antimicrobial activity against polymicrobial dentinal biofilms of common endodontic pathogens, such as *Staphylococcus aureus, Streptococcus mutans, Enterococcus faecalis*, and *Candida albicans* [42]. In addition, Neem extract showed significant anti-inflammatory activity in vitro [43]. Neem used in our experiments is commercial in form of capsules which contain 480 mg Neem leaf powder.

### 2.2. Thin Films Deposition

MAPLE is recognized as a suitable pulsed laser method for depositing polymers and other delicate substances in form of thin films, because it realistically replicates the characteristics and functionality of starting materials, without photochemical decomposition and damage under direct action of intense UV laser pulses [44,45,46,47,48]. 

First, a thin layer of PMMA was deposited by MAPLE on Ti and SS substrates. A second thin layer consisting of BG and Neem was applied by MAPLE onto the PMMA initial thin film. The resulting samples were denoted BGN/PMMA/Ti and BGN/PMMA/SS, as function of substrate material.

For the first MAPLE deposition, 0.6 g PMMA were dissolved into 19.3 mL chloroform to obtain a frozen target in liquid nitrogen [5]. For the second one, 0.12 g BG and 480 mg Neem were dissolved into 15 mL deionized water and frozen. 

The experiments were performed with a KrF* (λ = 248 nm, τ_FWHM_ ≤ 25 ns) excimer laser source operated at 5 Hz repetition rate and a fluence of 0.55 J/cm^2^. For the growth of each thin film, 5000 subsequent laser pulses were applied. In order to obtain a uniform layer and to avoid drilling, the substrate and target were simultaneously rotated at 50 rpm, while the background pressure inside the deposition chamber was set at 2 × 10^−2^ mbar. Throughout the depositions, the targets were kept at liquid nitrogen temperature using a cryogenic setting. The separation distance target to substrate was set at 4 cm.

### 2.3. Thin Films Characterization

#### 2.3.1. Morfological, Structural and Tribological 

The surface morphology of deposited films was inspected by scanning electron microscopy (SEM) with an FEI Inspect S electron microscope at 20 kV acceleration voltage in high vacuum using top-view and cross-section modes.

Prior to immersion in SBF, samples topography was scanned over a (15 × 15) µm^2^ area by Atomic force microscopy (AFM) using an TT-Workshop apparatus, in non-contact mode. Because of the high roughness of samples, the scanning speed was reduced to 0.5 Hz at 650 mV.

The stoichiometry and chemical functions integrity of thin films was investigated by Fourier-transform infrared spectroscopy (FTIR) and X-ray photoelectron spectroscopy (XPS). FTIR analysis was performed using a Nicolet ™ FT-IR iS ™ 50 spectrometer equipped with an ATR module, within (3700–700) cm^−1^ range at 4 cm^−1^ resolution. A Ge crystal with the following characteristics was used for ATR: 1.5 mm diameter, refractive index of 4 at 1000 cm^–1^, 0.67 μm penetration depth at 42° incidence and a spectral range between (5500–700) cm^–1^. The spectra were taken in absorbance mode. FT-IR spectra of single components were performed for Neem, BG and PMMA in powder form. XPS measurements were carried out in an ESCALAB Xi + (Thermo SCIENTIFIC Surface Analysis) configuration equipped with a multi-channel (dual source X-ray) electron beam analyzer that works with Al Kα (hv = 1486.2 eV) radiation, using C1s (284.4 eV) as an energy reference. Before data recording, the samples were degassed in the pre-chamber at a pressure of <2 × 10^−8^ Torr to remove the chemisorbed water on surface. Surface chemical compositions and oxidation states were estimated from XPS spectra using the Avantage software (version 5.978) using appropriate experimental sensitivity factors.

Tribological evaluation of nanostructures was conducted by Nano Scratch and Nano Tribometrical tests. The applied nano scratch-test method consists in the generation of scratches with a sphero-conical stylus which is drawn with a constant speed across the sample, under progressive loading at a fixed rate. The critical load (Lc) is defined as the smallest weight at which a detectable failure occurs. The driving forces for coating damage in the scratch test are in fact a combined effect of elastic-plastic indentation, frictional and residual internal stresses. Under the lower load regime, these stresses generally result in conformal or tensile cracking of the coating while still remaining fully adherent. The onset of these phenomena defines the first critical load. Under the higher load regime, one defines a critical load which corresponds to the onset of coating detachment from the substrate by spalling, buckling or chipping. This is the second critical load. Scratches have been performed on samples using the Nano Scratch Tester NST3 (Anton Paar, Austria). For a good statistic of the adhesion strength of the coating to the substrate, 4 × SS coated samples were analized. The samples were glued to the aluminum puck for analyses in air, at 37 °C and 34% humidity. The following test parameters were applied: sphero-conical indenter type, 5 µm indenter radius, 2 mN scanning load, 2 mN initial load, 100 mN final load, 294 mN/min loading rate, 1 mm scratch length, and 3 mm/min scratch speed. Lc1 is the normal load at which the first adhesion failure appears on either film or substrate (first cracks or first delamination of the coating) while Lc2 is the normal load at which the first chipping of the substrate occurs. 

It should be noticed that critical loads are widely regarded as indicators of the coating adhesion, while scratch tests serve as comparative methods. Indeed, critical loads are dependent on several parameters. They are either extrinsic (coating-substrate system) like substrate hardness, coating thickness, substrate and coating roughness, friction coefficient, or intrinsic (experimental conditions) as scratching speed, loading rate, diamond tip radius and diamond wear [49]. 

Tribometrical tests were performed using the Nano Tribometer NTR3 (Anton Paar, Austria) to measure the wear rate (friction coeficient) of samples. The samples were glued to an aluminum puck and analized in air, at 37 °C and 34% humidity. The wear rates were measured for each sample by 500 straight passes of a ruby ball with 4 mm diameter, 30 mN normal load, 0.3 cm/s maximum linear speed and 2 mm full amplitude. The result acquired at the middle of each cycle was considered the average value.

#### 2.3.2. Drug Release Behavior 

To study the insertion of implants into the human body and the phenomena occurring at the tissue-implant interface as a result of interaction with physiological fluids, coated Ti and SS substrates were immersed in 25 mL of simulated body fluid (SBF) in glass containers at 37 °C in a Binder Microbiological Incubator. They were investigated by FTIR and UV-VIS after different immersion times. 

SBF having an ionic composition identical to blood plasma was prepared according to Kokubo’s formula [50] by mixing the reagents following the specific order and quantity. Samples submerged in SBF were maintained at 37 °C using a microbiological incubator (Burning Microbiological Incubator (Drying oven)).

SBF solutions containing the release products from thin films were analysed by UV-VIS. The release profiles of Neem were recorded in absorbance mode by an Evolution 220 spectrophotometer (ThermoFisher Scientific, Darmstadt, Germany) within the range (190–1200) nm. All measurements were made in triplicate, in accordance with ISO / FDIS 23317: 2007 (E). The following protocol for 21-days immersion in SBF was applied: every 2 h in the first day, then after 1, 3, 7, 14 and 21 days of immersion. The absorption peaks amplitude, which is proportional to the drug concentration delivered by the implant to the surrounding tissue, was then determined.

#### 2.3.3. Electrochemical Investigation

Corrosion resistance of coatings was assessed via electrochemical methods. The influence of SBF on bare SS and coated substrates was studied by electrochemical impedance (EIS). Measurements were carried out using an electrochemical cell coupled with a PC-controlled frequency response analyzer (Palmsens, Utrecht, Nederlands). The data were acquired on the computer and processed with PcTrace software . A sinusoidal voltage with 0.01 V amplitude and 50,000 Hz to 0.1 Hz frequency was applied. Both EIS plots (the Nyquist diagrams) and Bode diagrams were recorded for each electrode under open circuit potential (OCP) configuration. They show features of the covering layer but also indicate the processes at liquid-solid interface. 

#### 2.3.4. Microbiological Assay 

Indirect cytotoxicity test was performed according to the International Standards BS-EN ISO 10993-12:2004 and ISO10993-5:2009 [51,52] to estimate biocompatibility of synthesized thin films to be implantated into the human body. 

Standard cell culture medium (Dulbecco's Modified Eagle Medium (DMEM) containing 1 mM sodium pyruvate, 10 mM (4-(2-hydroxyethyl)-1-piperazineethanesulfonic acid) (HEPES) buffer, 100 U/mL penicillin, 0.1 mg/mL streptomycin, 2 mM glutamine and supplemented with 10% (v/v) fetal bovine serum (FBS), hereafter referred as complete DMEM) served as the extraction vehicle. Briefly, material extracts were obtained by placing sterile BGN disk-shaped samples (Ø = 12 mm) in separated wells of 24-multiwell plates, and then 0.4 mL/well of complete DMEM was added. Samples were next incubated at 37 °C in a 5% CO_2_-humidified atmosphere for 1, 3 and 7 days (n = 3 samples/material/time step). At each time step, material extracts (eluates) were harvested and stored at –80 °C until use.

MG63 cells (human osteosarcoma cell line cells) (American Type Culture Collection, ATCC, Manassas, VA, USA) at passage 9 were cultured in complete DMEM at 37 °C in a 5% CO_2_-humidified atmosphere and used in tests. Cells were seeded in 96-multiwell plates at a density of 10^4^ cells/well in 100 μL of complete DMEM and incubated for 24 h under standard culture conditions (37 °C in a 5% CO_2_-humidified atmosphere). Next, the medium was replaced with 100 µL/well of eluates (n = 3 sample/wells /time step) and incubated for further 24 h under standard culture conditions. Cells cultured in complete DMEM were used as negative controls of cytotoxicity (-CTRL, n = 6), while cells cultured in 0.5% phenol solution in DMEM served as positive controls of cytotoxicity (+CTRL, n = 6).

To estimate the in-vitro biocompatibility, BGN/PMMA/SS samples with cells were placed into 24-well polystyrene plates and sterilized in pure ethanol for 30 min prior to examination. After 24 h of incubation, MG63 cells viability was assessed using AlamarBlue cell viability assay (Thermo Fisher Scientific, Monza, Italy). Briefly, the medium was removed and every well was loaded with 100 μL of specific medium containing 10 μL of resazurin dye solution. Cells were incubated under standard culture conditions for 2 h and the fluorescence of the medium was read by means of a GENios Plus reader (Tecan Group Ltd., Männedorf, Switzerland) (λ_ex_ = 540 nm; λ_em_ = 595 nm). 

Cell proliferation experiments were performed via fluorescence microscopy by growing SaOs2 cells both on samples surface and control (borosilicate glass). SaOs2 cells were selected for osteogenic cell differentiation due to a similar activity of alkaline phosphatase to that of primary osteoblasts. They produce high levels of core binding factor alpha1 (Cbfa1), Osterix (SP7), osteocalcin, bone sialoprotein, decorin and procollagen-I, and sustain enhanced mineralization [53].

The Ki67, a nuclear protein expressed in G1-S-M-G2 phases of the cell cycle and not in the G0 phase, was labelled for visualization in the green fluorescence channel. The cell’s nucleus was stained with 4’,6-diamino-2-phenylindole (DAPI) and visualized in the blue channel of the microscope BX51WI (Olympus, Tokyo, Japan). 

Time Kill method was used for the evaluation of the antimicrobial activity of samples by adapting and integrating the protocol described in ISO 22196 (Measurement of antibacterial activity on plastics and other non-porous surfaces) standards and ASTM E 2315 (Assessment of Antimicrobial Activity Using a Time-Kill Procedure). A small quantity of stock bacteria (*Staphylococcus aureus*, ATCC 29213 and *Escherichia coli* JM109, ATCC 25922, LTA S.R.L., Milano, Italy) was transferred from glycerol stock to a sterile Luria broth (LB) Agar plate (20 g/L of LB base - Invitrogen, Thermo Fisher Scientific, Monza, Italy—and 15 g/L of Agar (Invitrogen, Thermo Fisher Scientific, Monza, Italy) in distilled water) and incubated for 20 h at 37 °C. 

Before experiment, one single colony of bacteria was taken from the LBA plate and suspended in 5 mL of LB in a sterile polypropylene test tube and incubated overnight at 37 °C under shaking at 140 rpm. In the day of the experiment, the inoculum was prepared by diluting the bacteria to a starting OD600 = 0.02 in LB. Bacteria were let to grow until reaching OD600 ≈ 0.25–0.35, which corresponds to ≈ 1 × 10^8^ colony forming units (CFU)/mL. A total of 500 µL of bacteria suspension were next diluted 1:50 in sterile dH_2_O to a final concentration ≈ 1–2 × 10^6^/mL and inoculated on the surface of each sample. Samples were then incubated in darkness at 37 °C in a humidified incubator for 24 h. Then, an aliquot from each sample was taken and serially diluted in sterile dH_2_O and 50 µL were plated on P60 LB Agar plates. CFU were finally counted after overnight incubation at 37 °C. 

## 3. Results and Discussion

### 3.1. Surface Characterization of As-Deposited Structures

**Figure 1:**
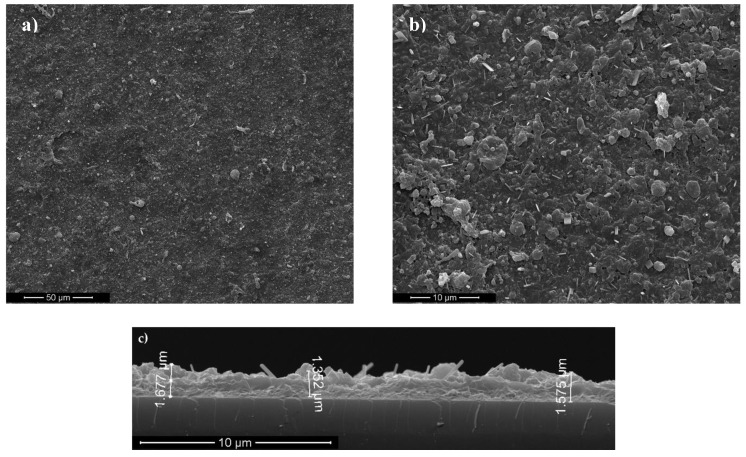
Typical SEM micrographs for BGN/PMMA coated substrates: (**a**) overview, (**b**) detail, (**c**) cross section.

Micro-topography of BGN/PMMA deposited thin films is visible from typical SEM images in Figure 1. One notices an uniform morphology with spherical, ovoid or elongated formations of variable dimension, characteristic to pulsed laser deposition methods [54]. The droplets are micrometric, randomly scattered throughout the surface (Figure 1a), and have, as known [55], a beneficial effect on cell adhesion and growth/proliferation. The high roughness of coatings is confirmed by the details in Figure 1b. Rod-like aggregates can be observed intercalated inside a specific laser deposition matrix. Taking into account the magnification and the software of the microscope, one can estimate the thickness of BGN/PMMA thin films of (1.53 ± 10%) µm (Figure 1c).

**Figure 2:**
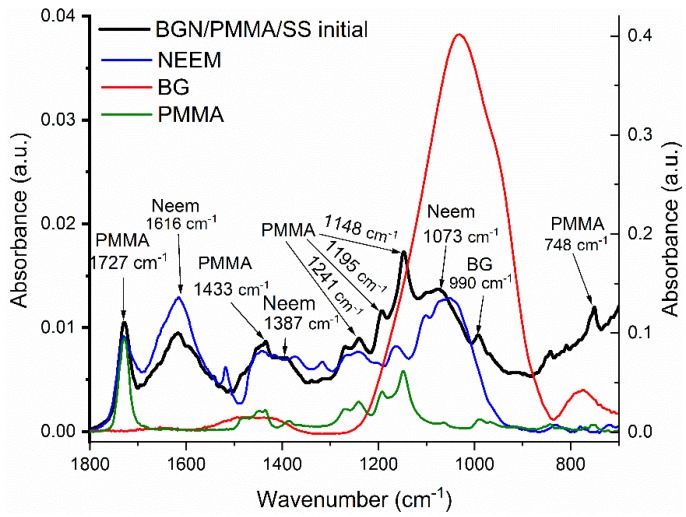
FTIR spectrum of BGN/PMMA/stainless steel (SS) film deposited by matrix-assisted pulsed laser evaporation (MAPLE) and of single components, namely Neem, BG, and PMMA.

Thin films preserve the composition of raw materials as demonstrated by corresponding FTIR spectra in Figure 2. Peaks belonging to BG, PMMA and Neem were identified. Thus, peaks attributed to the bending vibration modes of Si-O in BG (990 cm^−1^) [56] and to PMMA (at 1727, 1433, 1195, 1148 cm^−1^) [57,58] are clearly visible. As known, Neem is an organic compound with many components and molecular bonds, among which in Figure 2 are visible the folowing: (i) 1700–1500 cm^–1^ band, representative of N-H and C = O groups (aldehydes, ketones and esters); (ii) 1500–1350 cm^–1^ band, corresponding to the aromatic domain, especially N-H groups; and (iii) 1300–1500 cm^–1^ band, confirming the presence of alkenes [59,60].

XPS investigation (Table 1) revealed the presence of elements specific to BG (Si, Ca, P, Na) and Neem (C, O, N, P, Na).

**Figure 3:**
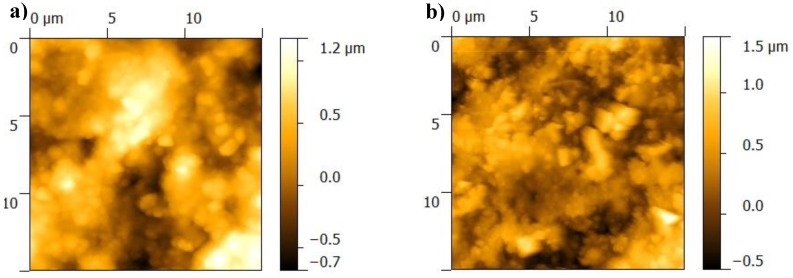
AFM images of films surface: (**a**) BGN/PMMA/Ti, (**b**) BGN/PMMA/SS.

From AFM analyses, one observes that the BGN/PMMA/Ti sample consists of large clusters of material separated by finer deposited areas (Figure 3). Clusters incorporate small particles, of about 100 nm diameter (Figure 3a)). There are larger clusters on surface of BGN/PMMA/SS sample (Figure 3b) than in case of BGN/PMMA/Ti structure. The particles embedded in clusters are lesser but larger, up to 300 nm diameter. AFM data (Table 2) are in good accordance with SEM observations regarding surface roughness and nanoparticles. 

**Figure 4:**
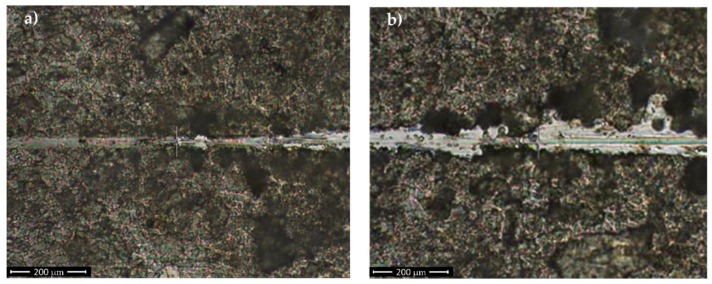
Innitial delamination (**a**) and first coating detachment (**b**) from SS substrate.

Progressive loading tests have been carried out on samples. One notices from Figure 4 that the force applied on the sample is not affected by the surface topography, probably due to the feedback loop control. The pre-scan procedure allows for measuring the actual penetration depth during scratch, while the characterization of the elastic recovery is performed via post-scan procedure. Each sample was tested three times and the average and standard deviation (SD) were calculated. The inferred average value of (165.14 ± 1.7) mN for critical load Lc1 and (250.23 ± 2.5) mN as the average value for critical load Lc2 are indicative for good coating adhesion and resistance to mechanical stress.

**Figure 5:**
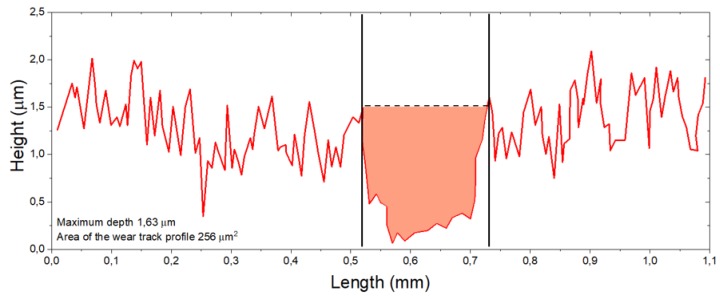
Profilometric measurement on BGN/PMMA/SS sample and mean wear track section (red field).

Four coated samples were three times tested under wet conditions in order to evaluate tribological properties. In Figure 5 are visible the profilometric measurement and mean wear track section (the red field) are visible. They were used to determine the radius and area of the wear track section and consequantly the loss of material volume (V) during the test. The material loss (Q), an important parameter which expresses the wear coefficient, can be inferred based on the formula:

$$Q=\frac{V}{F\cdot d}\;\left[mm^{3}/N\cdot m\right]$$

Here, F and d are the normal load and length on which the test was taken, respectively. The mean rate value of 1.1 × 10^−5^ mm^3^/(N.m) points to a good wear behavior of BGN/PMMA/SS samples.

The maximum penetration depth, as inferred from profilometric measurement, was of ~1.63 µm (Figure 5), in accordance with thickness estimation based upon the cross section SEM image (~(1.53 ± 10%) µm Figure 1c)). As deposited coating (Figure 4a)) exhibits a rather uniform morphology, in agreement with SEM micrographs (Figure 1a,c). 

### 3.2. Characterization of Structures after Immersion in SBF 

**Figure 6:**
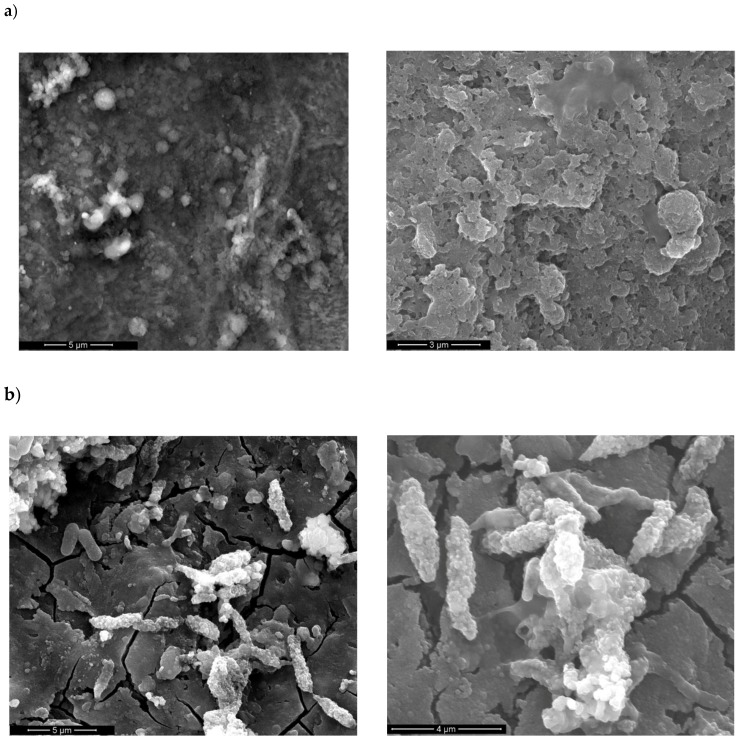
SEM images of (**a**) BGN/PMMA /Ti and (**b**) BGN/PMMA/SS thin films after 28 days of immersion in SBF, at different magnifications

As observed by SEM, BGN/PMMA films deposited on Ti (Figure 6a) and SS (Figure 6b) consist, after 28 days of immersion in SBF, of a rather smooth matrix covered with arbitrarily scattered micronic isolated or agglomerated spheroidal particles.

It the particular case of BGN/PMMA/SS thin films (Figure 6b), rod-like aggregates embedded into the deposition matrix are covered by particles of biological apatite, resulting from the BG–SBF interaction. This hypothesis is supported by energy-dispersive X-ray spectroscopy (EDS) results. Thus, a Ca/P molar ratio of ~1.30 was inferred and indicates the presence of biological apatite [54]. Trace elements characteristic to the bone mineral phase (Na, Mg, F) were detected besides the apatite-specific components (i.e., Ca, P and O) (data not shown). Elements characteristic to the SS substrate were also detected.

**Figure 7:**
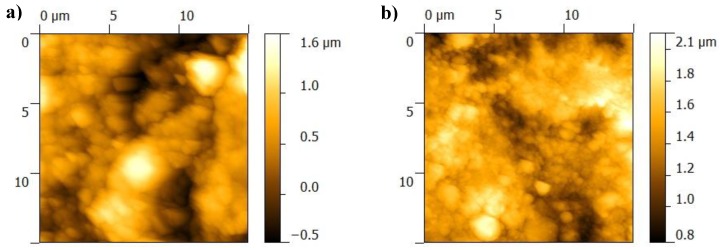
AFM images of thin films after immersion in SBF: (**a**) BGN/PMMA/Ti, (**b**) BGN/PMMA/SS.

Similar to SEM, AFM show areas with unevenly distributed particles on the surface (Figure 7). The topology is characterized by a RMS in the range (0.2–0.6) µm (Table 3). The higher values in case of samples deposited on Ti is not due to the non-equilibrium character of the depositing technique only, but to the formation of salts during immersion in SBF as well. 

The initial thin films display unevenly distributed particles on surface (compare Figure 3 and Figure 7). The surface morphology is changing with the soaking time in SBF: irregularities on surface get prominent , presumably because of BG dissolution and Neem release.

**Figure 8:**
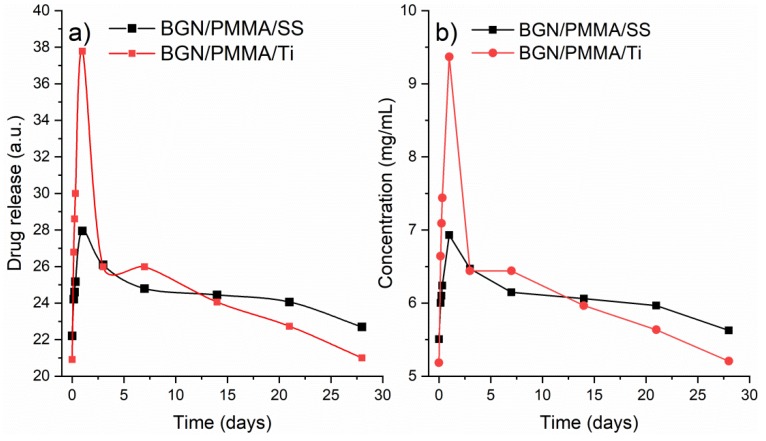
Drug release as function of time for BGN/PMMA/SS (black curve) and BGN/PMMA/Ti (red curve): (**a**) peak absorbance intensity function of time and (**b**) concentration function of time.

SBF containing products released from samples was analyzed by UV-VIS. The results are displayed in Figure 8 in both arbitrary units (a) but also as concentration variation in time (b). The trend in the two curves is similar. The maximum Neem amount (of 9.36 mg/mL for BGN/PMMA/Ti and of 6.93 mg/mL for BGN/PMMA/SS) released in all cases is reached within the first 24 h of immersion. Then, the release decreases steeply until the 7th day and thereafter slowly. It is of key importance to note that drug release still continues after 21 days. One may assume that the significant amount of drug released in the first day destroys the possibly adhered bacteria while, the smaller quantity of delivered drug over the following days prevents the formation of bacterial biofilm that can cause infections.

**Figure 9:**
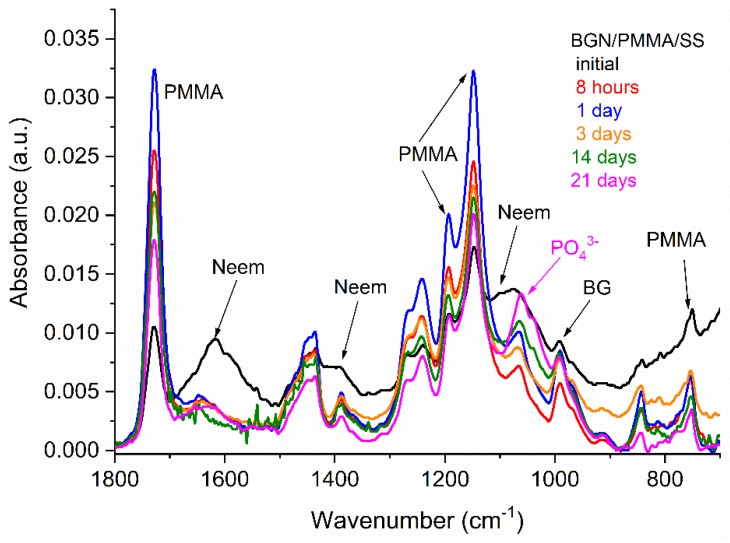
FTIR spectra of the initial and immersed samples for different time, respectively.

All samples were characterized by FTIR (Figure 9) according to the protocol in Section 2.3.2. Same features and settings of the apparatus as for FTIR evaluation of as-deposited samples were used for comparative reasons. 

One important observation is the persistence of Neem peaks after 21 days of immersion, which proves that the Neem delivery is not instantaneous but continuous.

Moreover, the FT-IR analysis, a qualitative characterization, was performed in order to support the quantitative characterization of UV-Vis analysis. Significant modifications of the FTIR spectrum are noticed even after 8 h of immersion (red curve in Figure 9). The bands at 1616 cm^−1^ and 1073 cm^−1^ belonging to Neem disappeared after 8 h of immersion in SBF suggesting its release. To our opinion, this is due to BG interaction with the surrounding liquid and eventual decomposition followed by Mg, Ca, K, Si, Na, P ions and Neem molecules release. 

Ions released from BG interact with SBF ions and a thin film of biological apatite starts growing on sample’s surface, as revealed by the advent of a new peak at 1060 cm^−1^ belonging to PO_4_^3−^ ions in hydroxyapatite, in accordance to SEM results. This evolution is typical for BG whenever immersed in SBF [29,30,31,32]. The new forming layer gradually covers the PMMA surface, as visualized by the attenuation of corresponding peaks in FTIR spectra. After the 14th day of immersion, a new band appeared at 1039 cm^−1^ suggesting the continuous formation of hydroxyapatite on the PMMA.

The FT-IR results are in accordance with the UV-Vis analysis showing the release of Neem in the SBF and more the covering of PMMA with hydroxyapatite.

**Figure 10:**
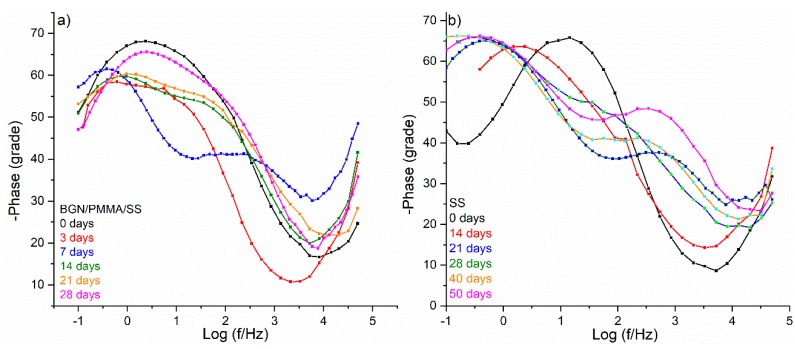
Bode diagrams for BGN/PMMA/SS (**a**) and SS (**b**) samples after different immersion time.

EIS was used to evaluate the evolution of layers under conditions simulating the biological interaction in human body (Figure 10). To this purpose, bare SS and BGN/PMMA/SS samples were immersed into 25 mL SBF and incubated at 37 °C. EIS spectra were recorded after 3, 7, 14, 21, and 28 days. Experiments were done in triplicate. 

Bode diagrams exhibit a very different appearance depending on the material and immersion time. Both one time and two time constant diagrams are displayed in Figure 10. The maximum phase angle with major significance for the evaluation of involved physical-chemical processes was determined (Table 4). The as-deposited BGN/PMMA/SS sample has a one time-constant diagram (68.14° max phase angle), indicative for a well capacitive BGN/PMMA protective layer. Two peaks are present on Bode diagram after three days of immersion. The one at 53.41° suggests the BG dissolution and release of its components as well as of Neem, in accordance with FTIR analysis. After 7, 14, and 21 days of immersion, the Bode diagrams conserve a similar behaviour. It means that this structure acts as a long-term drug delivery system, with great potential against biofilm formation. After 28 days of immersion, the single 65.60° max phase angle in Bode diagram suggests the formation of a new hydroxyapatite layer covering and shielding the sample, as also observed by FTIR. 

A completely different behavior was noticed in the case of bare SS sample (Figure 10b and Table 4). The initial max phase angle of 65.68° slowly decreases after immersion, pointing to a fairly stable surface. After 21 days of immersion, the sample presents two-time constants and Bode diagrams have two peaks. The one at 47.39° points to an intense corrosion in SBF. The process continues in this way for long time, so the behaviour of SS is the same even after 40 or 50 days of immersion. SS is inappropriate for use as implant material, because its behaviour can stay at the origin of aseptic loosening inside human body [11]. However, these undesired effects can be removed by our strategy for coverage of SS implant with BGN/PMMA thin protective layers, when a suitable behaviour in SBF was observed.

**Figure 11:**
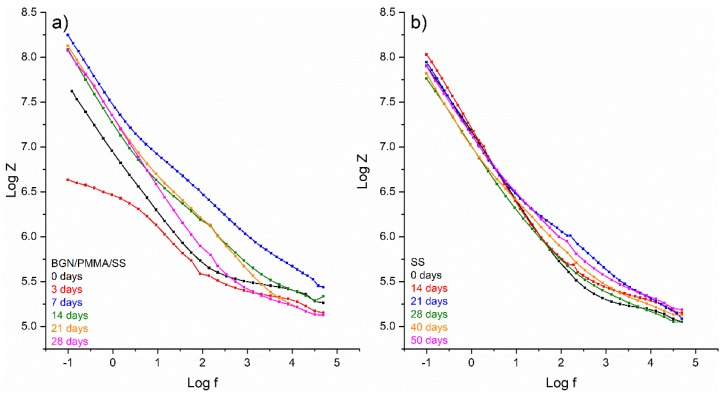
Impedance plots for BGN/PMMA/SS (**a**) and SS (**b**) samples after different immersion time.

Impedance Log Z (log f) plots evidenced a completely different behavior: a continuous, slow, long-time change of substrate surface as result of SS degradation into SBF, and a sudden, short-time modification in BGN/PMMA/SS sample, respectively. The BGN/PMMA/SS structure acts similarly with an ideal capacitor (black curve in Figure 11a). After 28 days it finishes as a pure capacitor with modified electrical resistance and capacitance [61]. 

### 3.3. Biological Assays 

**Figure 12:**
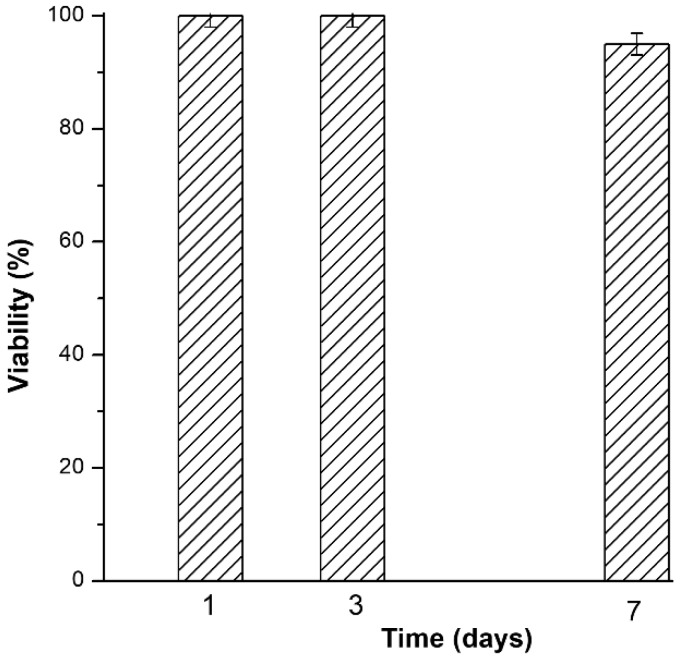
Cytotoxicity test results.

Figure 12 shows the fraction (%) of viable MG63 cells after 24 h of incubation with eluates of BGN obtained at different time steps (1, 3 and 7 days of extraction process). Viability of -CTRL cells was estimated as 100%, while viability of +CTRL was found 0% corresponding to a drastic cytotoxic effect. This means that deposited nanostructures are friendly materials in contact with cultivated cells, in particular MG63.

**Figure 13:**
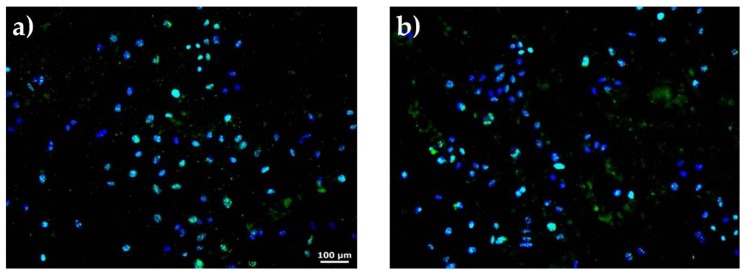
Fluorescence microscopy images of SaOs2 cells labeled with Ki67 and DAPI after cultivation on BGN/PMMA/SS (**a**) and SS (**b**) samples. 100X magnification.

SaOs2 cells labelled with both DAPI and Ki67 were counted. We performed five determinations for each sample and compared with the control (cells grown on borosilicate glass) (Figure 13). The data are expressed by: (number of Ki67 positive cells / number of cells labelled with DAPI) × 100.

**Figure 14:**
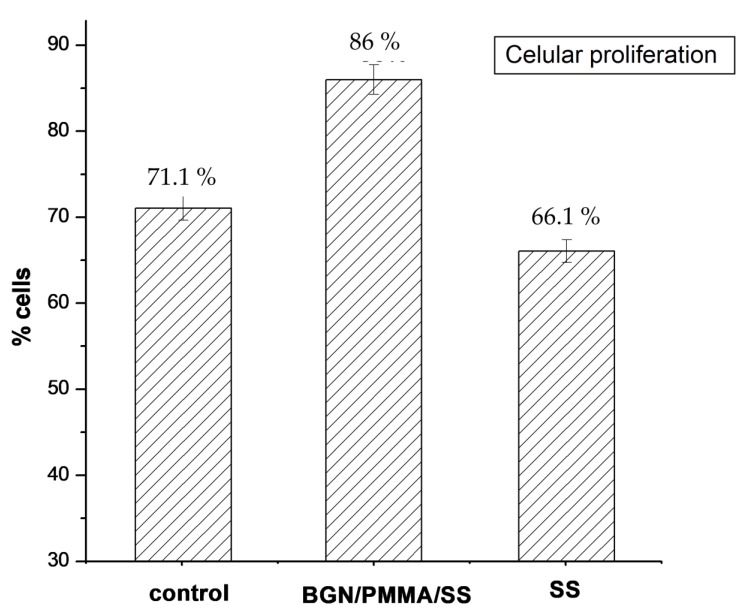
Proliferation of cells at passage 9 after 6 days from seeding on BGN/PMMA/SS and bare SS samples.

As visible from Figure 14, osteoblasts seeded on BGN/PMMA/SS films proliferated in a larger proportion (86%), superior to cells in the control experiment (71.1%) or SS samples (66.1%).

On the other hand, antimicrobial activity of BGN/PMMA/SS and bare SS was evaluated over 24 h after incubation with *Staphylococcus aureus* and *Escherichia coli*. Antimicrobial activity of BGN/PMMA/SS is strong against Gram positive bacteria (*S. aureus*) but rather poor in respect with Gram negative bacteria (*E. coli*). Indeed, BGN/PMMA/SS displays a 95.1% antibacterial efficiency on *S. aureus* but only about 38.2% on *E. coli*, as visible from Table 5. It should be emphasized that BGN/PMMA/SS exhibits a generally higher antibacterial activity as compared to bare SS.

## 4. Conclusions

A local drug release system is projected to be an alternative to prevent or to treat bone infections associated with implants. Our concept is based on a functional bioapatite—biopolymer double nanostructure which controls delivery of natural antimicrobial drug extracts. *Azadirachta indica* (Neem) was selected because of the proved antibacterial efficacy of its extracts against most frequent microorganisms causing osseous or dental infections: *Staphylococcus aureus* and *Escherichia coli,* respectively.

A thin double-layered structure was fabricated by MAPLE to cover a metallic (Titanium or stainless steel) implant. All structures were studied prior and after 1, 3, 7, 14 and 21 days of immersion in SFB at 37 °C. Deposited structures were covered by isolated or clustered particles embedded in a typical laser deposition matrix, as shown by SEM and AFM. EDS, FTIR and XPS investigations proved that MAPLE allows a congruent transfer of elements from target to an appropriate implant-like substrate. 

Our study demonstrated a rapid osseointegration and enhanced corrosion resistance of the covered bioinert metallic implant. It was shown to prevent the release of metal ions in the body, concomitant with a notable antimicrobial activity by gradual drug release at implantation site.

## Figures and Tables

**Table 1:** Elemental distribution on BGN/PMMA/SS sample surface.

Name	Peak BE	FWHM (eV)	Area (P) CPS.eV	Atomic %
C 1s	284.10	3.57	604263.05	56.74
O 1s	531.21	304	814956.33	29.49
Si 2p	101.08	2.77	50090.55	4.91
N 1s	398.97	2.82	37012.54	2.17
Ca 2p	346.22	2.45	131256.80	2.11
P 2p	132.01	2.63	4006.47	0.26
Na 1s	1071.8	5.36	13990.24	0.21

**Table 2:** Roughness values of thin films before immersion in simulated body fluid (SBF).

Sample	RMS (µm)	Ra (µm)
BGNPMMA/Ti	0.331	0.268
BGN/PMMA/SS	0.272	0.219

**Table 3:** The roughness values of analyzed thin films after immersion in SBF.

Sample	RMS (µm)	Ra (µm)
BGN/PMMA/Ti	0.630	0.537
BGN/PMMA/SS	0.357	0.281

**Table 4:** Max phase angle for BGN/PMMA/SS and SS samples after different immersion time.

Sample	Time (days)	Max Phase Angle (grade)	
**BGN/PMMA/SS**	0	68.14	
	3	58.53	53.41
	7	61.46	41.15
	14	59.80	53.76
	21	60.55	54.14
	28	65.60	
**SS**	0	65.68	
	14	63.53	
	21	66.00	47.39
	28	65.02	37.52
	40	65.68	41.65
	50	66.16	48.23

**Table 5:** Antibacterial activity of the samples against Staphylococcus aureus and Escherichia coli.

Bacteria (CTRL)	Studied Samples	Bacteria vs CTRL (% mean ± st. dev.)	Antibacterial Efficiency (% vs CTRL)
*Staphylococcus aureus*	BGN/PMMA/SS	5.1 ± 1.2	95.1
	SS	7.9 ± 2.5	79.0
*Escherichia coli*	BGN/PMMA/SS	59.1 ± 3.8	38.2
	SS	64.5 ± 4.6	30.9
